# Delayed motherhood: Birth outcomes at extremely advanced maternal age: A retrospective cohort study

**DOI:** 10.1097/MD.0000000000047017

**Published:** 2026-01-16

**Authors:** Alaattin Karabulut, Sercan Kantarci, Uğurcan Dağli, Elif Atici, Alper İleri, Defne Engür, Özgün Uygur, Volkan Karataşli

**Affiliations:** a Department of Obstetrics and Gynecology, University of Health Sciences Tepecik Training and Research Hospital, Bornova, Izmir, Turkey; b Department of Neonatology, University of Health Sciences Tepecik Training and Research Hospital, Izmir, Turkey; c Department of Gynecologic Oncology, Balikesir Atatürk City Hospital, Balikesir, Turkey.

**Keywords:** advanced maternal age, cesarean delivery, neonatal outcomes, perinatal complications

## Abstract

This study aimed to evaluate and compare pregnancy and neonatal outcomes in women aged ≥ 45 years, categorized as having extremely advanced maternal age (EAMA), and a control group of women aged 25 to 35 years who delivered within the same time period. A retrospective cohort study was conducted at a tertiary referral center in Turkey, including 143 women aged ≥ 45 and 250 women aged 25 to 35 who gave birth between January 2017 and January 2024. Obstetric and neonatal outcomes were compared, including gestational age, mode of delivery, birthweight, Apgar scores, and neonatal intensive care unit (NICU) admissions. Cesarean delivery (71.3% vs 47.6%, *P* < .001), preterm birth (21.0% vs 9.2%, *P* = .001), and NICU admissions (21.7% vs 12.8%, *P* = .02) were significantly more frequent in the EAMA group. The median birthweight was also significantly lower in this group (3000 g vs 3305 g; *P* < .001), and extremely preterm births (<28 weeks) occurred only in the EAMA group. Women aged ≥ 45 years face significantly higher risks of adverse obstetric and neonatal outcomes, underscoring the need for tailored prenatal care and delivery planning in this growing patient population as delayed motherhood becomes increasingly prevalent.

## 1. Introduction

The trend of delayed childbearing has become increasingly prevalent worldwide, particularly in developed and developing countries, including Turkey. Factors such as increasing female participation in higher education and the workforce, access to assisted reproductive technologies (ART), and shifting sociocultural norms have contributed to rising maternal age at first birth. Consequently, births among women aged 45 years and older – defined as extremely advanced maternal age (EAMA) – are now encountered more frequently in obstetric practice, albeit still representing a small proportion of total births.^[1–3]^

According to several studies, women 45 years of age and older had greater rates of gestational diabetes mellitus, hypertensive disorders, cesarean delivery, abnormal placentation, preterm birth, and admission to the neonatal intensive care unit (NICU) than women in younger age groups.^[2–6]^ Fewer studies clearly isolate the EAMA group, and the majority of the material now in publication concentrates on women over the ages of 35 or 40.

The Turkish Statistical Institute reports that in 2024, women aged ≥45 accounted for just 0.26% of all births, with over 60% of births occurring in the 25 to 35 age bracket.^[7]^ Because of the disproportionately high risks of complications in this population, EAMA’s clinical impact is nevertheless substantial despite its low prevalence.

This retrospective cohort study seeks to examine and compare pregnancy and neonatal outcomes between women aged ≥45 years and a randomly selected group of women aged 25 to 35 years, who delivered within the same seven-year timeframe (January 2017 to January 2024). This study aims to add to the body of information guiding counseling and management in advanced maternal age pregnancies by comparing the EAMA cohort to a low-risk group and provide clinically significant insights. Notably, unlike most studies that use ≥35 or ≥40 years as thresholds for advanced maternal age, our research explicitly focuses on women aged ≥45 years, a group that has rarely been isolated in previous investigations. This provides novel insights into the specific risks encountered by this understudied population. Furthermore, presenting data from a middle-income country such as Turkey enhances the study’s relevance, as sociocultural factors, health system differences, and varying access to ART may significantly influence pregnancy outcomes compared with high-income settings.

## 2. Methods

This retrospective cohort study was conducted at the University of Health Sciences Tepecik Training and Research Hospital, Department of Obstetrics and Gynecology, a tertiary referral center in Turkey. Ethical approval for the study was obtained from the Tepecik Training and Research Hospital Ethics Committee (approval number: 2024/09-23). The study was conducted in accordance with the principles of the Declaration of Helsinki. The authors declare that there are no conflicts of interest related to this research.

The study included women who gave birth between January 2017 and January 2024. The following were excluded: multiple pregnancies, maternal alcohol or tobacco use during pregnancy, intrauterine fetal death, fetal abnormalities, births before 20 weeks of gestation, or birthweights under 500 grams. Women 45 years of age and older who gave birth throughout the study period (n = 143) made up the study group. The hospital information management system was used to identify these cases. The control group comprised 250 women aged between 25 and 35 years who delivered during the same period. Control subjects were selected randomly from a pool of over 35000 eligible births using a computer-generated simple random sampling method. This age range was chosen to represent the optimal reproductive age, and the control sample size was determined based on similar studies^[2]^ and statistical power calculations to allow detection of differences in adverse outcomes.

Delivery-related parameters such as gestational age at birth, mode of delivery, birthweight, 1-minute and 5-minute Apgar scores, and the need for NICU admission were recorded for all participants. Gestational age at delivery was categorized as preterm (<37 weeks), very preterm (<32 weeks), extremely preterm (<28 weeks), and postterm (>42 weeks). Birthweight was classified into 4 groups: low birth weight (<2500 grams), very low birth weight (<1500 grams), extremely low birth weight (<1000 grams), and macrosomia (>4000 grams).

All data were obtained from the hospital’s electronic medical records. Statistical analyses were conducted using SPSS version 22.0 (IBM Corp., Armonk). The Kolmogorov–Smirnov test was used to assess normality of continuous variables. Parametric data were presented as mean ± standard deviation and compared using the independent samples *t*-test. Non-parametric data were expressed as median (interquartile range) and compared using the Mann–Whitney *U* test. Categorical variables were analyzed using the chi-square test. A *P*-value < .05 was considered statistically significant.

## 3. Results

A total of 393 women were included in the analysis: 143 in the EAMA group (≥45 years) and 250 in the control group aged between 25 and 35 years.

The rate of cesarean delivery was significantly higher in the EAMA group compared to the control group (71.3% vs 47.6%, *P* < .001). Preterm delivery (<37 weeks of gestation) occurred more frequently in the EAMA group (21.0%) than in the control group (9.2%) (*P* = .001). Moreover, extremely preterm births (<28 weeks) were observed only in the ≥45 years group (2.1%, n = 3), which was statistically significant (*P* = .04) (Table 1).

**Table 1:** Perinatal outcomes according to maternal age.

Perinatal outcomes	25–35 yr, n = 250, n (%)	≥45 yr, n = 143, n (%)	*P*-value*
Caesarean delivery	119 (47.6)	102 (71.3)	<.001†
Extremely preterm (<28 wk)	0 (0)	3 (2.1)	.04†
Very preterm (<32 wk)	7 (2.8)	4 (2.8)	>.99†
Preterm (<37 wk)	23 (9.2)	30 (21)	.001†
Postterm (>42 wk)	1 (0.4)	1 (0.7)	>.99†

* Significant at .05 level.

† Chi-square test.

Median birthweight was significantly lower in the EAMA group compared to the control group (3000 g vs 3305 g; *P* < .001). NICU admission was significantly more common among neonates born to women in the EAMA group (21.7% vs 12.8%, *P* = .02) (Table 2).

**Table 2:** Neonatal outcomes according to maternal age.

Neonatal outcomes	25–35 yr, n = 250, n (%)	≥45 yr, n = 143, n (%)	*P*-value*
Extremely low birth weight (<1000 g)	1 (0.4)	3 (2.1)	.13†
Very low birth weight (<1000–1500 g)	3 (1.2)	4 (2.8)	.26†
Low birth weight (1500–2500 g)	26 (10.4)	18 (12.6)	.50†
Macrosomia (>4000 g)	25 (10)	7 (4.9)	.07†
NICU admission	32 (12.8)	31 (21.7)	.02†
Low Apgar at 1st min (<7)	22 (8.8)	21 (14.7)	.09†
Low Apgar at 5th min (<7)	8 (3.2)	7 (4.9)	.39†
Birthweight (g; median, 25%–75%)	3305 (2761–3695)	3000 (2640–3390)	<.001‡

NICU = neonatal intensive care unit.

* Significant at .05 level.

† Chi-square test.

‡ Mann–Whitney *U* test.

## 4. Discussion

This study shows that women who are 45 years old or older are much more likely to have adverse perinatal outcomes than women who are 25 to 35 years old. These results are in line with previous research that shows that maternal age (≥45 years) is a significant risk factor for complications during pregnancy and infancy.

One of the most striking findings we found in our study was that older mothers had a much higher rate of cesarean delivery (71.3% vs 47.6%, *P* < .001). Earlier studies found similar results, which said that the higher rate was due to both mothers asking for it more often and the fact that this age group has more pregnancy-related problems, like labor dystocia, fetal growth restriction, or preeclampsia. In addition to medical indications, cultural factors and clinical practices in Turkey may contribute to the elevated cesarean delivery rates observed in the ≥45 age group. Maternal request, increased caution among clinicians when managing pregnancies in older mothers, and institutional policies regarding delivery practices are all likely to influence these outcomes.^[1,2,8–11]^

The birthweight distributions of the 2 groups were also significantly distinct. Along with higher rates of low and very low birthweight, the median birthweight was significantly lower in the ≥45 age group. These results might be the result of decreased placental function or higher prevalences of comorbid conditions like diabetes or gestational hypertension, which are more common in older pregnancies.^[2,6,12]^ The EAMA group also had less favorable neonatal outcomes. The higher NICU admission rate (21.7% vs 12.8%) and lower Apgar scores indicate increased neonatal morbidity. This is consistent with earlier research that found a higher risk of neonatal resuscitation, preterm birth, and the need for intensive care is associated with older mothers.^[2,3,13]^ Significantly more preterm births occurred in the ≥45 group, and only in this cohort did extremely preterm deliveries occur. In line with other cohort studies and population-based registries, these results emphasize the influence of maternal age on uteroplacental function and the probability of an early delivery. Several underlying mechanisms may explain the higher rates of adverse outcomes in EAMA pregnancies. Maternal comorbidities such as chronic hypertension, diabetes mellitus, and cardiovascular disease are more prevalent in this population, and these conditions may impair placental perfusion and oxygen transfer, leading to intrauterine growth restriction and preterm delivery. In addition, age-related uteroplacental insufficiency, endothelial dysfunction, and vascular rigidity can compromise placental development and function. Reduced ovarian reserve and alterations in endocrine pathways, including impaired hormonal milieu, may further contribute to suboptimal placentation and fetal growth restriction. Furthermore, diminished physiological reserves and the cumulative effects of oxidative stress and chronic inflammation in older mothers may increase susceptibility to complications during gestation. Collectively, these biological processes provide a plausible explanation for the lower birthweights, increased NICU admissions, and higher rates of very preterm births observed in our cohort.^[3,5,11,14]^

Fewer studies have isolated the ≥45 subgroup, despite the fact that prior research frequently compares younger populations to those with ≥35 or ≥40 age thresholds. By directly comparing this age group with women in the optimal reproductive range (25–35 years), our findings provide more detailed insights into the specific risks that this growing population faces, especially given delayed childbearing and increased use of ART.^[2,4]^

The limitations of this study include its retrospective design and being conducted at a single center, which may reduce the generalizability of the findings. Another limitation of this study is the lack of long-term neonatal follow-up, which prevents evaluation of potential developmental or health consequences beyond the perinatal period. Future studies incorporating longitudinal neonatal and childhood outcomes would provide a more comprehensive understanding of the implications of EAMA on offspring health.

In light of these findings and consistent with prior literature, broader clinical and public health implications emerge regarding the management of pregnancies in this age group. According to this retrospective cohort study, compared to women in the optimum reproductive age range of 25 to 35 years, pregnancies among women 45 years of age and older are substantially linked to higher risks of unfavorable obstetric and neonatal outcomes. The EAMA group had lower median birthweights, low Apgar scores, preterm birth, cesarean delivery, and NICU admissions. These results highlight how crucial it is for older pregnant women to receive tailored preconception counseling, closer antenatal monitoring, and delivery planning. Through multidisciplinary and risk-focused perinatal care, healthcare systems must adjust to the unique clinical challenges presented by this population as maternal age at childbirth continues to rise globally.

## Author contributions

**Conceptualization:** Alaattin Karabulut.

**Data curation:** Alaattin Karabulut, Elif Atici, Defne Engür, Özgün Uygur.

**Formal analysis:** Alaattin Karabulut.

**Investigation:** Alaattin Karabulut.

**Methodology:** Alaattin Karabulut.

**Project administration:** Alaattin Karabulut.

**Resources:** Uğurcan Dağli.

**Software:** Uğurcan Dağli.

**Supervision:** Alper İleri.

**Validation:** Sercan Kantarci.

**Visualization:** Sercan Kantarci.

**Writing – original draft:** Alaattin Karabulut.

**Writing – review & editing:** Alaattin Karabulut, Volkan Karataşli.
